# SAXS analysis of a soluble cytosolic NgBR construct including extracellular and transmembrane domains

**DOI:** 10.1371/journal.pone.0191371

**Published:** 2018-01-18

**Authors:** Joshua Holcomb, Maysaa Doughan, Nicholas Spellmon, Brianne Lewis, Emerson Perry, Yingxue Zhang, Lindsey Nico, Junmei Wan, Srinivas Chakravarthy, Weifeng Shang, Qing Miao, Timothy Stemmler, Zhe Yang

**Affiliations:** 1 Department of Biochemistry and Molecular Biology, Wayne State University School of Medicine, Detroit, Michigan, United States of America; 2 Department of Pharmaceutical Sciences, Wayne State University, Detroit, Michigan, United States of America; 3 Center for Synchrotron Radiation Research and Instrumentation and Department of Biological and Chemical Sciences, Illinois Institute of Technology, Chicago, Illinois, United States of America; 4 Department of Surgery, Children’s Research Institute, Medical College of Wisconsin, Milwaukee, Wisconsin, United States of America; Universitetet i Bergen, NORWAY

## Abstract

The Nogo-B receptor (NgBR) is involved in oncogenic Ras signaling through directly binding to farnesylated Ras. It recruits farnesylated Ras to the non-lipid-raft membrane for interaction with downstream effectors. However, the cytosolic domain of NgBR itself is only partially folded. The lack of several conserved secondary structural elements makes this domain unlikely to form a complete farnesyl binding pocket. We find that inclusion of the extracellular and transmembrane domains that contain additional conserved residues to the cytosolic region results in a well folded protein with a similar size and shape to the *E*.*coli* cis-isoprenyl transferase (UPPs). Small Angle X-ray Scattering (SAXS) analysis reveals the radius of gyration (R_g_) of our NgBR construct to be 18.2 Å with a maximum particle dimension (D_max_) of 61.0 Å. *Ab initio* shape modeling returns a globular molecular envelope with an estimated molecular weight of 23.0 kD closely correlated with the calculated molecular weight. Both Kratky plot and pair distribution function of NgBR scattering reveal a bell shaped peak which is characteristic of a single globularly folded protein. In addition, circular dichroism (CD) analysis reveals that our construct has the secondary structure contents similar to the UPPs. However, this result does not agree with the currently accepted topological orientation of NgBR which might partition this construct into three separate domains. This discrepancy suggests another possible NgBR topology and lends insight into a potential molecular basis of how NgBR facilitates farnesylated Ras recruitment.

## Introduction

The Nogo-B receptor (NgBR) plays an essential role in angiogenesis during wound healing and embryonic development [1–4]. In wound healing angiogenesis, NgBR is bound by Nogo-B ligand that activates Akt pathway to stimulate endothelial cell chemotaxis [2]. During embryonic development, NgBR is essential for blood vessel assembly, and knockout of NgBR results in embryonic lethality due to severe defects in vasculature development, protein glycosylation and cholesterol homeostasis [3, 4]. NgBR was identified as an essential subunit of dolichol biosynthetic machinery that has a well-defined role in the early stages of protein N-glycosylation [5]. NgBR binds and stabilizes Nieman-Pick type C2 protein (NPC2) to regulate cholesterol homeostasis and intracellular cholesterol trafficking [6]. NgBR is also involved in several cancers. It is highly expressed in the late stages of invasive ductal carcinoma and human hepatocellular carcinoma [7, 8]. NgBR promotes the epithelial-mesenchymal transition of breast tumor cells and contributes to the chemo-resistance of hepatocellular carcinoma [7, 8]. However, the underlying mechanism of NgBR in tumor cell growth is still poorly understood.

Recently, we showed that NgBR is involved in breast cancer cell growth through modulating Ras signaling [9]. NgBR promotes the accumulation of Ras at the plasma membrane and distributes the activated Ras to non-lipid-raft membrane. Knockdown of NgBR causes diminished Ras accumulation and inhibits EGF-stimulated Ras signaling and tumorigenesis. NgBR serves as a docking site for Ras plasma membrane accumulation and directly binds to farnesylated Ras. The farnesylation of the C-terminal CAAX box of Ras is sufficient for NgBR binding. These suggest that NgBR may directly recognize the farnesyl group for promoting Ras membrane localization and its subsequent interaction with regulators and effectors. NgBR shares a high sequence similarity to the cis-isoprenyl transferase family (cis-IPtase) [5]. This protein family was found to bind and catalyze condensation of isopentenyl diphosphate to farnesyl diphosphate (FPP). In the case for FPP interaction, binding of the farnesyl group is mediated by a deep hydrophobic pocket. NgBR maintains essential residues responsible for forming this conserved hydrophobic pocket [5, 9]. This suggests that NgBR and the cis-IPtase family may share a similar structural mechanism in binding to a farnesyl group.

The structure of NgBR is currently unknown. Previous circular dichroism (CD) spectroscopy and NMR data showed that the cytosolic domain of NgBR is only partially folded and its extracellular region intrinsically disordered [10]. It also remains elusive about its transmembrane topology. NgBR is a transmembrane protein containing 293 amino acids. Three transmembrane topologies have been proposed. At the endoplasmic reticulum (ER) membrane, NgBR has three transmembrane regions with the C-terminal domain either in cytosol or ER lumen [5]. At the plasma membrane, NgBR was considered as a type I single pass transmembrane protein with the C-terminal domain located in the cytosol [1, 10]. Though significantly different, these topologies share a common transmembrane region (residues 120–139) and the same C-terminal domain (residues 140–293). This C-terminal domain shares most sequence similarity to cis-IPtase and was proposed to interact with NPC2 in the ER lumen, the cytosolic region of mammalian cis-IPtase (hCIT) at the ER membrane, and farnesylated Ras at the plasma membrane [5, 6, 9]. However, in this study, our structure and sequence analysis suggests that the C-terminal domain alone is insufficient to form a farnesyl binding pocket due to the lack of a number of conserved structural components. This is consistent with the C-terminal domain alone being partially folded. Based on additional sequence similarity, we designed a protein construct that includes residues corresponding to the entire cytosolic domain of NgBR, its transmembrane domain and some extracellular components. Interestingly, this construct consisting of residues 79–293 is soluble and monodispersed. Small angle X-ray scattering (SAXS) analysis shows the construct is well folded with a similar shape and size to the *E*.*coli* cis-IPtase. The similar radius of gyration (R_g_), Porod volume, and D_max_ indicate NgBR folds as a single globular domain in solution as the cis-IPtase does. However, this result conflicts with the previously proposed topology of NgBR at the plasma membrane which might partition this construct into three separate domains. This conflict raises a possibility of the presence of another topological orientation for NgBR.

## Materials and methods

### Protein expression and purification

NgBR was essentially expressed and purified as previously described [11, 12]. In brief, the open reading frame corresponding to human NgBR (residues 79–293 or 137–293) was amplified and cloned into a pCDF-SUMO vector containing an His_6_-SUMO tag. The vector was transformed into BL21(DE3) cells for recombinant protein expression. Cells were inoculated into LB media and grew to an optical density of 0.5. Cells were then induced with 0.1 mM isopropylthio-β-D-galactoside (IPTG) and grew at 15°C overnight. After harvest, cells were lysed using a French Press, and the supernatant was collected for purification. After the His_6_-SUMO tag was cut by yeast SUMO protease 1, the native NgBR(79–293) was separated from the tag by a second Ni^2+^ column. Finally, NgBR was purified by a size exclusion column (Hiload 16/60 Superdex 200, GE Healthcare) in 20 mM HEPES pH 7.5, 150 mM NaCl, 5% glycerol and 1 mM Tris(2-carboxyethyl)phosphine (TCEP).

### Small angle X-ray scattering data collection and analysis

Small angle X-ray scattering (SAXS) data was collected at BioCAT beamline at Argonne National Laboratory (S1 File). Samples consist of 20 mg/mL NgBR(79–293) in the buffer containing 20 mM HEPES pH 7.5, 150 mM NaCl, 5% glycerol, 1 mM TCEP. Scattering measurements were collected at 25°C using a 100 μL capillary flow-cell. To separate different oligomer states, samples were subjected to scattering analysis in-line with size-exclusion chromatography (Superdex 200 10/300 GL, GE Healthcare). Scattering frames corresponding to monomeric NgBR were scaled, averaged and subtracted from averaged buffer frames. Scattering data was analyzed using the ATSAS suite [13]. Radius of gyration (R_g_) was calculated using the Guinier approximation. Pair distribution function (*P(r)*) and the maximum particle dimension (D_max_) were estimated from the scattering data using the GNOM algorithm. An *ab initio* dummy atom model was generated using DAMMIN [14]. The molecular weight (MW) of the model was estimated by taking the volume from the model and dividing it by 2 [14]. The theoretical scattering curve of the UPPs (undecaprenyl pyrophosphate synthase) crystal structure (PDB code: 1X08) [15] was calculated with CRYSOL [16]. The R_g_ and D_max_ of the UPPs were also calculated with CRYSOL. The Porod volume of the UPPs were estimated from the theoretical *P(r)* calculated from the CRYSOL simulated data.

### Circular dichroism

Circular dichroism measurements were carried out with an JASCO J-1500 Circular Dichroism spectrophotometer (JASCO, Easton, MD). Spectra were collected from 185 nm to 260 nm at 20°C using a 0.01 mm pathlength cuvette with 4.0 mg/ml NgBR, 5 mM potassium phosphate buffer (pH 7.4), 1 mM 2-Mercaptoethanol. The spectra reported represent an average of 50 consecutive scans with the background signal from the buffer subtracted. Data was normalized and expressed as the mean residue ellipticity ([Θ]_MRE_) in the unit of deg.cm^2^.dmol^-1^. The percentages of the secondary structure elements were estimated from the spectra using BESTSEL [17]. The secondary structure contents of UPPs (PDB code: 1X08) were calculated using STRIDE [18].

## Results

### A soluble cytosolic construct including extracellular and transmembrane domains

**Fig 1:**
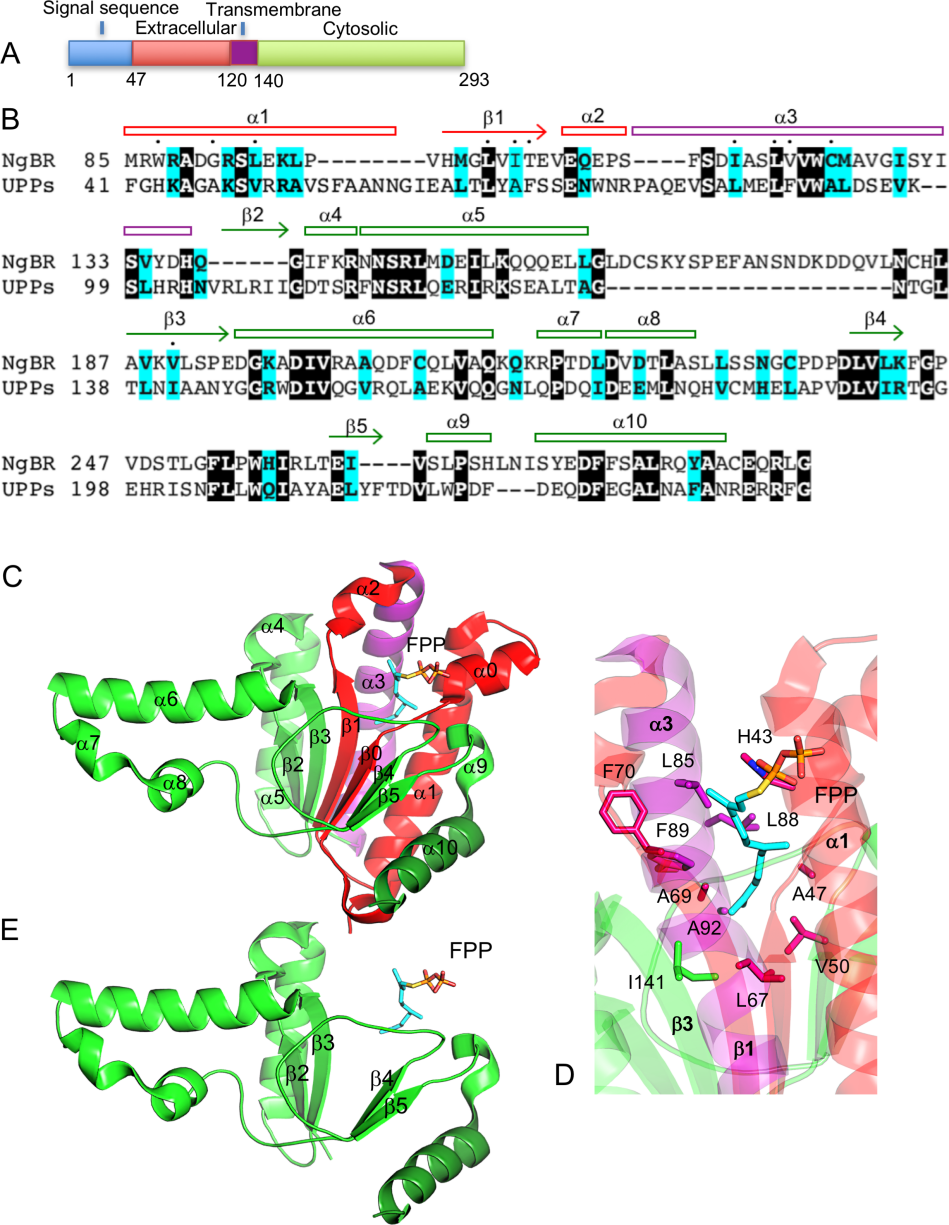
NgBR shares high sequence similarity with UPPs. (A) Topological structure of NgBR at the plasma membrane. Domains are colored according to their topological location. Extracellular, red; transmembrane, purple; cytosolic, green. (B) Sequence alignment of NgBR and UPPs. Identical residues are represented as white on black and similar residues are colored in cyan. Residues involved in binding farnesyl pyrophosphate (FPP) are indicated by dots. Secondary structures are numbered based on their sequence position. (C) Ribbon diagram of UPPs structure (PDB code: 1X08). The structure is colored according to the corresponding NgBR domains. Coloring scheme same as 1A. The secondary structural elements are labeled according to 1B. Bound FPP is depicted as sticks. (D) FPP binding pocket in UPPs. (E) A model of UPPs without structural elements corresponding to the extracellular and transmembrane regions of NgBR.

**Fig 2:**
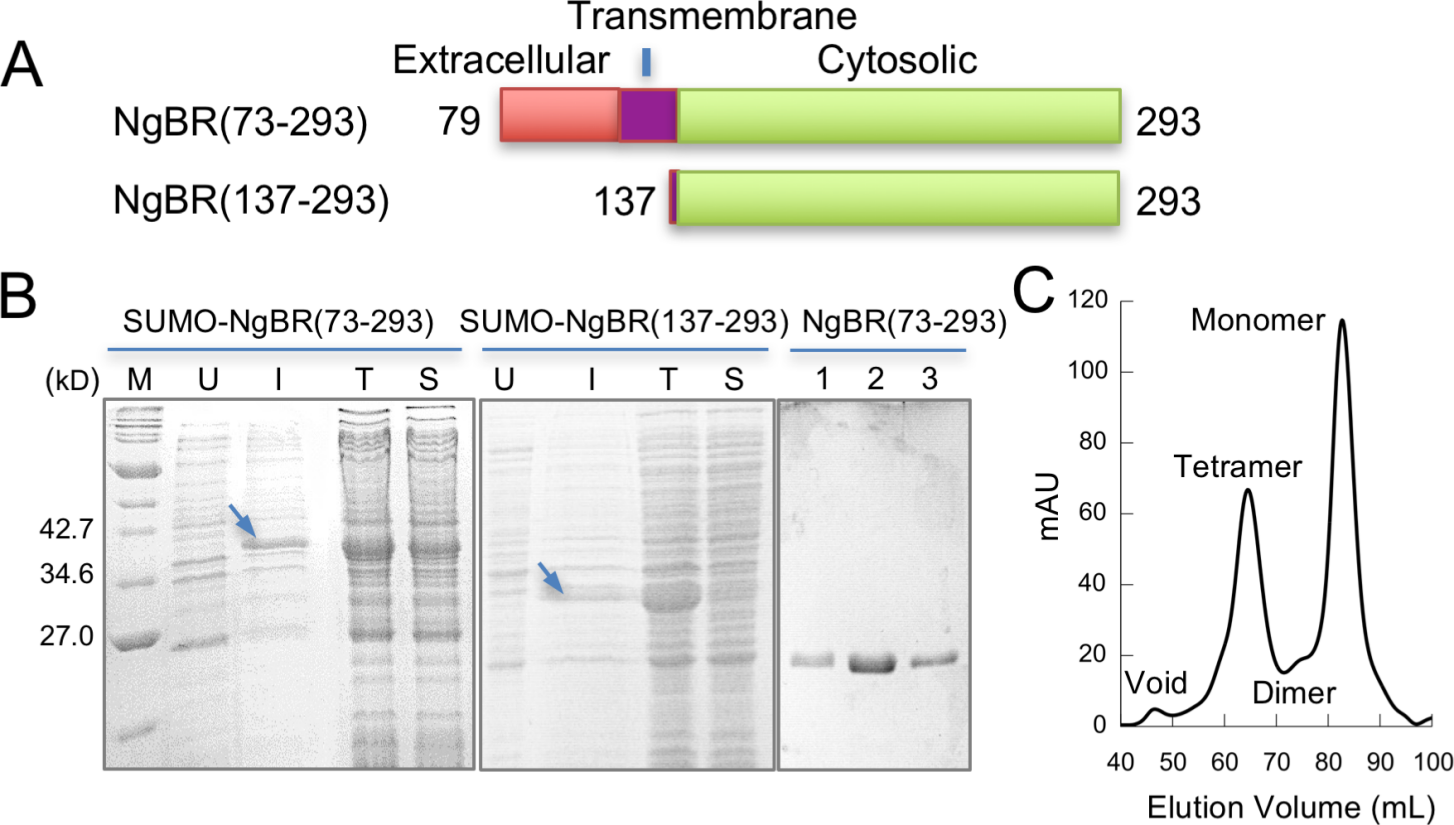
A soluble and monodispersed NgBR construct. (A) NgBR protein constructs. (B) SDS-PAGE analysis of NgBR protein expression and purification. SUMO-NgBR(73–293), left; SUMO-NgBR(137–293), middle; purified NgBR(73–293), right. Lane M, molecular weight marker; U, uninduced cell culture; I, induced cell culture; T, total cell lysate; S, supernatant of cell lysate; 1–3, different monomeric fractions. (C) Elution profile of NgBR(73–293) size-exclusion chromatography.

NgBR was considered as a type I transmembrane protein at the plasma membrane [10]. It consists of a putative 46-residue signaling sequence at the N-terminus, an extracellular domain containing residues 47–119, a single transmembrane region spanning residues 120–139, and a cytoplasmic domain consisting of residues 140–293 (Fig 1A). NgBR shares 24% sequence identity and 39% sequence similarity to the *E*.*coli* cis-IPtase undecaprenyl pyrophosphate synthase (UPPs) (Fig 1B). This level of sequence similarity suggests a similar structural fold and farnesyl binding mode between NgBR and UPPs. UPPs folds as a triangle-shaped structure with a central six-stranded parallel β-sheet packed by seven parallel α-helices on one side and three perpendicular protruding α-helices on the other (Fig 1C). The farnesyl binding site is formed between the parallelly packed α-helices and β-sheet involving α1, α3, β1, and β3. The bound farnesyl group interacts with the residues His43, Ala47 and Val50 from α1, Leu67, Ala69 and Phe70 from β1, Leu85, Leu88, Phe89 and Ala92 from α3, and Ile141 from β3 (Fig 1D). These residues together form a deep hydrophobic pocket recognizing a stretched farnesyl conformation. However, the C-terminal domain of NgBR alone lacks the majority of the farnesyl-interacting residues and only contains β3 (Fig 1B). Without α1, α3, and β1, the farnesyl binding pocket would not be intact, and there would be structural gaps between the rest of the secondary structural elements (Fig 1E). This is consistent with our protein expression data and previous NMR data that the C-terminal domain alone has low solubility and is only partially folded (Fig 2) [10]. However, this incomplete farnesyl binding pocket is very likely unable to bind a farnesyl group, which does not agree with the fact that NgBR binds to farnesylated Ras in the cytosol [9].

Additional sequence similarity is present in NgBR transmembrane domain and some extracellular regions (Fig 1B). The transmembrane domain can be aligned with the helix α3 of UPPs. The extracellular region from residues 79–119 can be aligned with α1 and β1. Therefore, at the primary sequence level, NgBR contains all secondary structural elements for the formation of a farnesyl binding pocket, and over 90% of the residues involved in the farnesyl binding in UPPs is conserved in NgBR. This suggests that NgBR could form a complete farnesyl binding pocket if its transmembrane domain and some of its extracellular regions are present in the cytosol. Although this hypothesis does not agree with the current topological orientation of NgBR at the plasma membrane (Fig 1A), it is supported by the fact that some conserved residues in the transmembrane domain are required for NgBR to bind farnesylated Ras [9]. Residues Ile117 and Leu120 of the transmembrane domain correspond to the farnesyl-interacting residues Leu85 and Leu88 from α3 of UPPs (Fig 1B and 1D). Mutation of these two residues to an alanine significantly reduced the interaction of NgBR with farnesylated Ras [9]. This suggests that the transmembrane domain of NgBR might have structural and functional roles analogous to the helix α3 of UPPs that contributes to an intact farnesyl binding pocket and overall folded structure (Fig 1C).

Based on the sequence alignment, we designed a NgBR protein construct (residues 79–293) that contains all conserved residues for NgBR to form a complete, UPPs-like structural fold and farnesyl binding pocket (NgBR(79–293)). The construct includes the entire cytosolic region, the transmembrane domain and some extracellular regions (Fig 2A). The construct was cloned and expressed in *E*.*coli*. Over 50% of total recombinant NgBR present in the cell lysis was recovered in the supernatant fraction (Fig 2B). In contrast, a construct including only NgBR cytosolic region (residues 137–293) is largely insoluble (Fig 2A and 2B). This indicates that inclusion of the additional residues, i.e., the extracellular region and transmembrane domain, in the prior construct enhances the recombinant protein solubility. In addition, the construct can be purified to homogeneity using chromatography (Fig 2B and 2C). The size-exclusion chromatography revealed four distinct peaks, with the majority of proteins corresponding to NgBR in the monomeric state.

### Small angle X-ray scattering and circular dichroism analysis

**Fig 3:**
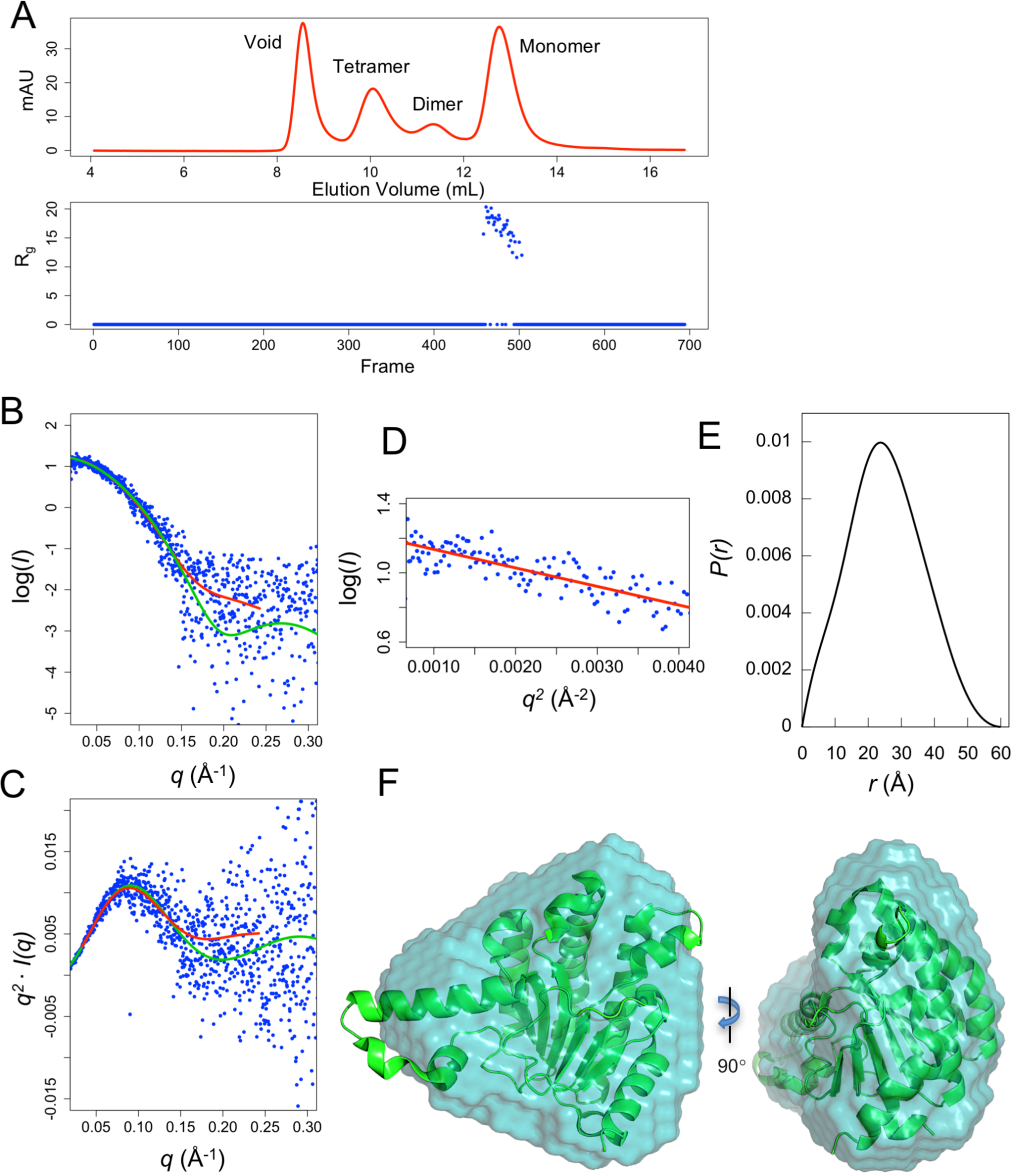
SAXS analysis of monomeric NgBR(73–293). (A) In-line SEC-SAXS. The elution profile of NgBR size-exclusion chromatography (top) aligns with the Rg plot of SAXS frames (bottom). (B) Experimental scattering curve of NgBR (blue dots) overlaid with the theoretical scattering curve calculated from UPPs structure (green, χ^2^ = 1.61) and an *ab initio* dummy atom model (red, χ^2^ = 1.31). (C) Kratky plot of NgBR (blue) overlaid with UPPs theoretical curve. (D) Guinier plot. (E) Pair distance function of NgBR scattering. (F) An *ab initio* dummy atom model of NgBR (surface) superimposed with UPPs crystal structure (ribbon) (PDB code: 1X08).

NgBR(79–293) needs to fold into a single globular domain in order to form a structural fold that can have a farnesyl binding pocket similar to that of UPPs. However, if it adopts the currently proposed topology, this construct might not fold into a single globular domain, and might be partitioned into three separate regions. To determine the fold states of NgBR(79–293), Small Angle X-ray Scattering (SAXS) was performed with the purified protein. SAXS is a method that provides information on protein size, shape and fold states, and can distinguish between single-domain globularly-folded proteins and multi-domain proteins with flexible linkers and intrinsically disordered regions. Thus, SAXS analysis of NgBR should be able to reveal the fold states of NgBR in solution. To ensure monodispersity of the sample, NgBR(79–293) was subjected to synchrotron SAXS in-line with size-exclusion chromatography. Concentrated NgBR consisting of monomer, dimer, and tetrameric states were separated by size-exclusion chromatography followed by in-line X-ray scattering on the elution from the column (Fig 3A). Only the monomeric state can be analyzed due to a low protein concentration and aggregation of the higher oligomer states. The scattering curve for monomeric NgBR follows the theoretical scattering of UPPs (PDB code: 1X08) very well at low *q* range with a slight variation found at increasing *q* values (Fig 3B). This indicates that NgBR may exhibit an overall structure similar to that of UPPs but with some higher resolution structural differences. The Kratky plot of NgBR scattering demonstrates a similar pattern with low *q* values of NgBR following closely to UPPs theoretical scattering and slight variation at high *q* values (Fig 3C). Notably, the overall curve of NgBR Kratky plot indicates a well folded protein in solution. This is demonstrated by a bell-shaped peak at low *q* which converges to the baseline at high *q*, in contrast to that a plot will not converge to the baseline if a protein is partially or completely disordered, and that there are additional peaks at low *q* if the protein contains multiple domains [19].

Further analysis of the SAXS data confirms that NgBR has a similar overall size and shape to UPPs (PDB code: 1X08). The radius of gyration (R_g_) value for NgBR is 18.2 Å which closely coincides with the R_g_ of UPPs (18.6 Å) (Fig 3D). The maximum particle dimension (D_max_) of NgBR is 61.0 Å similar to UPPs D_max_ of 58.6 Å. Analysis of NgBR pair distribution function reveals a bell-shaped peak which is characteristic of a globularly folded protein (Fig 3E). The Porod volume of NgBR is calculated to be 37, 200 Å^3^ similar to 36, 800 Å^3^ of UPPs. Finally, *ab initio* shape modeling reveals the molecular envelope structure of NgBR in solution with an estimated molecular weight (MW) of 23.0 kD (Fig 3F). This is consistent with the calculated MW for our NgBR construct (24.1 kD). In addition, the NgBR molecular envelope exhibits a triangular shape which superimposes well with the UPPs crystal structure (Fig 3F). Altogether, this demonstrates that the recombinant monomeric NgBR protein consisting of residues 79–293 folds as a single globular domain in solution with an overall size and shape similar to UPPs.

**Fig 4:**
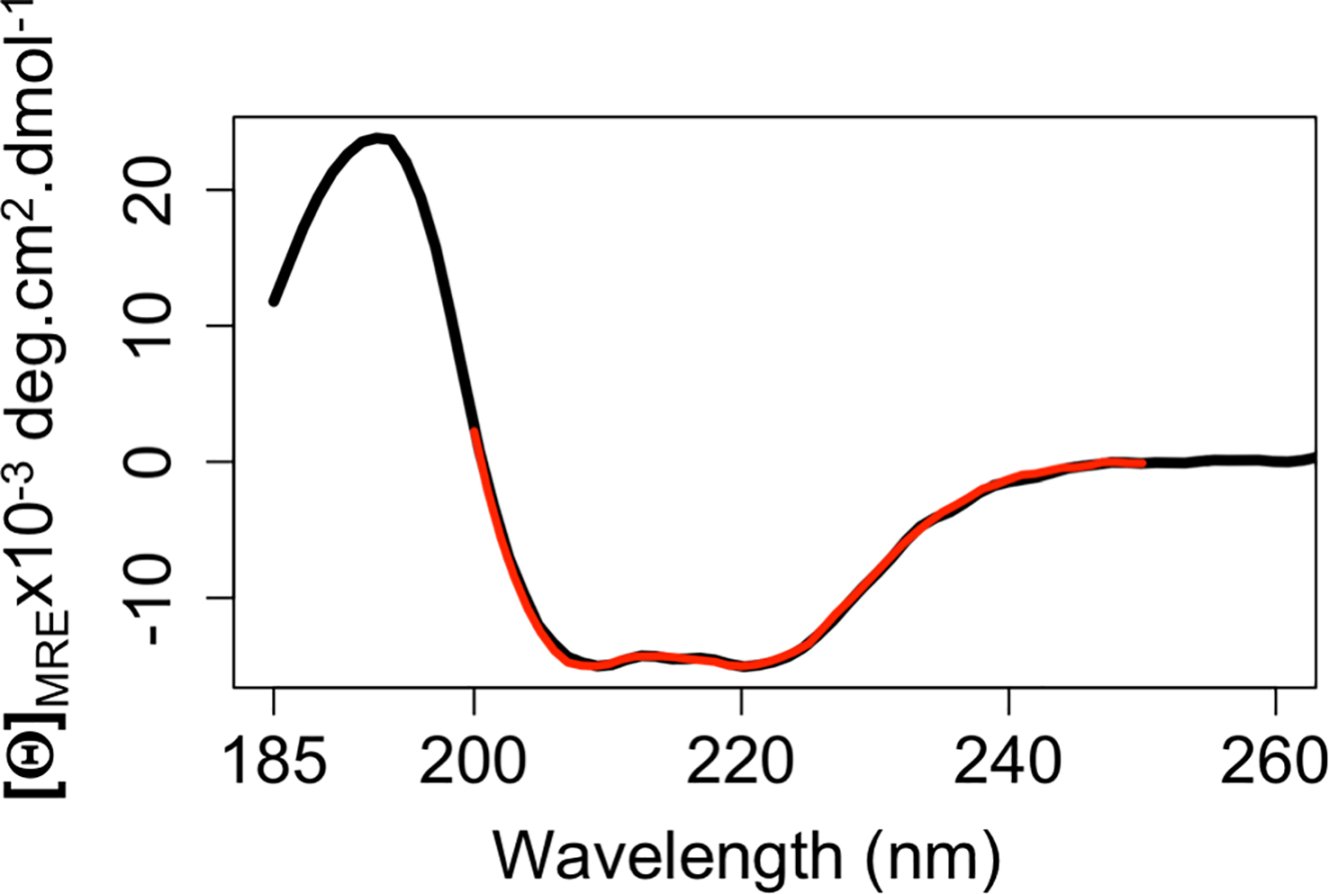
Circular dichroism analysis of monomeric NgBR(79–293) with the mean residue ellipticity (black) fit using the program BESTSEL (red). The fitting NRMSD (normalized root-mean-square deviation) is 0.016.

To further determine that NgBR(79–293) is folded, the secondary structure of this construct was probed by circular dichroism (CD) spectroscopy. The spectra reveal a strong broad negative band between 200 nm and 235 nm (Fig 4). The lowest points of the spectra are found at 209 nm and 222 nm. This indicates an α-helix dominant structure. The percentage of α-helix is estimated to be 48.3% from the spectra and β-strand 14.7%. This is consistent with the percentages of α-helix (47.0%) and β-strand (13.4%) of the UPPs (PDB code: 1X08). This further suggests that NgBR(79–293) is folded in solution with the secondary structure contents similar to the UPPs.

## Discussion

The high sequence similarity between NgBR and the farnesyl-binding protein cis-IPtase suggests that these two proteins may share a similar overall structural fold and farnesyl binding mode. However, the cytosolic region of NgBR itself is insufficient and unlikely to form a complete farnesyl binding pocket due to the lack of several conserved secondary structural elements. The recombinant construct of the cytosolic region is only partially folded and largely insoluble. However, we show that inclusion of the extracellular and transmembrane domains that contain additional conserved residues to the cytosolic region can significantly enhance the recombinant protein solubility. SAXS analysis of this protein construct demonstrates that it forms a single globular domain in solution with an overall size and shape similar to *E*.*coli* cis-IPtase. This construct is well folded without any evidence of structural disorder or characteristics of a multi-domain protein. However, our data appears to be in disagreement with the currently proposed topological orientation of NgBR which might separate this construct into three domains: cytosolic, transmembrane and extracellular. One possible explanation for this discrepancy is that NgBR can adopt another topological conformation and the construct NgBR(73–293) may represent a cytosolic region of a new, not-yet-identified topology.

NgBR has been proposed to adopt several topological conformations [1, 5, 10]. At the ER membrane, the orientation of the C-terminal domain towards the cytosol and ER lumen was defined by trypsin sensitivity in digitonin permeablizied cells and endoglycosidase H sensitivity [5]. At the plasma membrane, the N-terminal residues from 64–74 was found extracellularly using a polyclonal antibody specific to this region and the C-terminus intracellularly using an antibody against a C-terminal engineered tag [1]. However, the transmembrane topology of NgBR in both studies was predicted using the transmembrane-region prediction algorithms. While each such topology needs to be thoroughly confirmed by further experiments, whether and how NgBR can undergo topological transition and domain translocation between different conformational topologies are unknown. Multiple cases have been observed where membrane proteins adopting varying topological conformations can undergo domain translocation due to alteration in the phospholipid environment [20–23]. In the case of *E*.*coli* LacY where deficiency of phosphatidylethanolamine results in the inversion of its N-terminal six-transmembrane helical bundle and translocation of its seventh transmembrane domain to the extracellular space [20, 21]. A similar topological transition is observed in the case of phenylalanine permease (PheP) upon phosphatidylethanolamine reduction. In this case, PheP is able to undergo complete inversion of its N-terminal first helical hairpin [24]. With evidence of such domain translocation, it may be possible that NgBR experiences a similar change with the internalization of the extracellular and transmembrane regions to form a UPPs-like structural fold and farnesyl binding pocket upon a physiological stimulus. While further experiments should confirm if NgBR can exist in such a topological orientation, our study opens an avenue for additional structural characterization of NgBR and lends insight into a potential molecular basis of how it facilitates farnesylated Ras recruitment and promotes oncogenic Ras signaling.

## Supporting information

S1 File Small angle X-ray scattering (SAXS) frames. (ZIP)
